# Efficacy and Safety of Oral Cloxacillin Versus Amoxicillin-Clavulanic Acid in Treating Pediatric Acute Bacterial Lymphadenitis

**DOI:** 10.7759/cureus.81963

**Published:** 2025-04-09

**Authors:** Akhil D Sai, Utkarsh Bansal, Nivedita P Yerramilli, Vijay Singh, Venkat S Kumar, Smriti Ahuja, Girjesh K Singh

**Affiliations:** 1 Pediatrics, Hind Institute of Medical Sciences, Barabanki, IND

**Keywords:** antibiotic resistance, bacterial lymphadenopathy, cervical lymphadenopathy, cervical lymph node, cloxacillin, co-amoxiclav, fine needle aspiration cytology (fnac), lymphadenopathy, lymph node, visual assessment score

## Abstract

Aim and objectives

Acute bacterial lymphadenitis is one of the most common causes of lymphadenopathy in children, whose treatment algorithm is not well defined in literature to date. The most commonly used drug for this condition is amoxicillin-clavulanic acid. However, given escalating antibiotic resistance, the use of narrower-spectrum agents like cloxacillin could be preferred. Thus, this study was planned to compare the safety and efficacy of oral cloxacillin and amoxicillin-clavulanic acid in pediatric bacterial lymphadenitis.

Materials and methods

This was a cohort analytical study done on children aged 1-14 years presenting with acute onset of lymphadenopathy. After establishing a bacterial etiology, the patients were randomized to receive either oral amoxicillin-clavulanic acid (Group A) or cloxacillin (Group B) and followed up as per protocol. The primary outcome was the duration of defervescence from the initiation of treatment. Secondary outcomes were a reduction in lymph node size, tenderness, leukocytosis, erythrocyte sedimentation rate (ESR), and C-reactive protein (CRP).

Results

A total of 192 patients were diagnosed with acute bacterial lymphadenitis and randomized into Group A (n = 99) and Group B (n = 93). Subjective and objective parameters obtained in both groups were compared before and after the initiation of treatment. The mean defervescence time for Group A was 5.92 days (95% confidence interval (CI): 5.15 to 6.69 days) and for Group B was 5.74 days (95% CI: 4.96 to 6.52 days). The upper bound of 95% CI in Group B (6.52) was only 0.5 days longer than the mean of Group A (5.92). This finding suggests the non-inferiority of cloxacillin in comparison with amoxicillin-clavulanic acid.

Conclusion

Oral cloxacillin is as effective and safe as compared to amoxicillin-clavulanic acid in uncomplicated pediatric bacterial lymphadenitis.

## Introduction

Lymphadenopathy is defined as a change in the number, size, and consistency of one or more lymph nodes [1]. Acute bacterial lymphadenitis, caused by *Staphylococcus aureus* and *Streptococcus pyogenes* in 40% to 80% of cases, is one of the most common causes of lymphadenopathy in the pediatric age group [2]. Acute lymphadenitis (≤2 weeks) most commonly affects the cervical lymph node group and is concerning to both the child and the parents. Subacute (two to six weeks) and chronic (≥6 weeks) lymphadenitis more often involves varied lymph node groups, tends to be generalized, and is usually seen in older children and adolescents [1,3].

The treatment algorithm for this condition is not well defined in literature, often leading to confusion regarding the choice of antibiotic. Both amoxicillin-clavulanic acid and cloxacillin have a good gram-positive coverage and are active against *S. aureus*, which is one of the most common organisms associated with acute bacterial lymphadenitis [4]. Both drugs are commonly used in clinical practice, but due to broader coverage, many clinicians favor the use of amoxicillin-clavulanic acid [5]. However, given the rising rates of antibiotic resistance, it may be preferable to use narrower-spectrum agents like cloxacillin as the first line and reserve the broader-spectrum amoxicillin-clavulanic acid for resistant cases [6]. There is a paucity of studies comparing the efficacy of different antibiotics in acute bacterial lymphadenitis. We hypothesized that cloxacillin is non-inferior to amoxicillin-clavulanic acid in the treatment of acute uncomplicated bacterial lymphadenitis in children and conducted a study to compare both drugs in terms of efficacy and safety.

## Materials and methods

This prospective analytical study was conducted at Hind Institute of Medical Sciences, located in the Barabanki district of Uttar Pradesh, between June 2023 and November 2024. During the study period, all children, of either sex, between the ages of one and 14 years who presented with lymphadenopathy were enrolled. Informed and written consent was taken from parents and assent from the children above seven years of age. The ethics clearance was obtained before the commencement of the study from the Institutional Ethical Committee. A detailed history was taken and a local examination of the lymph node was done. A baseline visual assessment score (VAS) was noted for objective assessment of pain. Investigations done included complete blood count (CBC), C-reactive protein (CRP), erythrocyte sedimentation rate (ESR), and fine needle aspiration cytology (FNAC).

The procedure of FNAC was done by the pathology team under all aseptic precautions by using a 22 G needle and a 10 mL disposable plastic syringe. Needle aspiration was done under ultrasound guidance depending on the proximity of the enlarged node to vital neurovascular structures. The aspirated contents were then transferred onto a clean slide, and smears were prepared. The wet smears were stained with hematoxylin and eosin (H&E) and the air-dried smears with May-Grunwald-Giemsa (MGG) stain. They were then examined and reported by the pathology team in our institute.

All patients with acute (≤2 weeks) or subacute (two to six weeks) onset lymphadenopathy with associated clinical signs of underlying inflammation with CBC showing neutrophilic leukocytosis and elevated inflammatory markers like CRP and/or ESR and FNAC showing reactive changes were included in the study. Children with a history of chronic onset (>6 weeks) or known allergy to the penicillin group of drugs or concomitant chronic systemic illnesses (congenital heart disease, chronic respiratory disease, malabsorption syndromes, chronic liver disease, and chronic kidney disease) or complicated disease requiring injectable antibiotics and/or surgical interventions were excluded. All children who satisfied the inclusion criteria were randomized into two groups (Group A and Group B) by a computer-generated randomization table, using simple randomization done by MS Excel (Microsoft Corporation, Redmond, WA, United States) by applying the appropriate algorithm. They received amoxicillin-clavulanic acid (Group A) at a dose of 50 mg/kg/day every 12 hours for 10 days or cloxacillin (Group B) at a dose of 50 mg/kg/day every six hours for 10 days. Blinding in any form could not be done due to the obvious differences in the dosing schedule of the two drugs.

Parents were provided with a digital thermometer and a temperature chart and were instructed to measure and record the axillary temperature every eight hours on the temperature chart. During the follow-up on days 5, 10, and 14, the symptoms and signs were reviewed. The VAS was re-assessed on day 5, investigations were repeated on day 10, and the side effect profile of the drugs was inquired on days 5, 10, and 14. The primary outcome was the duration of defervescence from the initiation of treatment. Secondary outcomes were a reduction in lymph node size, tenderness, leukocytosis, ESR, and CRP.

The sample size was calculated by considering the efficacy of amoxicillin-clavulanic acid as 94.4% [7] and assuming a margin of 15% non-inferiority for cloxacillin. For a significance level (α) of 0.05, statistical power (1-β) of 80%, and an allocation ratio of 1:1, the sample size calculated was 92 in each study group using the MedCalc software (MedCalc Software Ltd., Oostende, Belgium) [8]. Assuming an attrition of 15% during follow-up, the final sample size was 105 in each study group. The data were compiled in Microsoft Excel. Descriptive variables were analyzed using the Statistical Package for the Social Sciences (SPSS) software 22.0 (released 2013; IBM Corp., Armonk, NY, United States). Categorical variables were expressed as percentages (%), while quantitative variables were presented as the mean and standard deviation (SD) or median and interquartile range (IQR). For paired comparisons, the independent t-test was employed. The chi-squared test was utilized for intergroup comparison of categorical variables. All analyses were two-tailed, and a p-value < 0.05 was deemed statistically significant.

## Results

During the study period, a total of 336 patients with lymphadenopathy were encountered in the pediatric outpatient department (OPD). Out of these, 72 (21.42%) had baseline investigations suggestive of and FNAC-proven tuberculosis, nine (2.67%) had a diagnosis of malignancy, and 15 (4.46%) had an unknown etiology and were excluded from the study. Thirty-three (9.82%) had FNAC suggestive of reactive changes but were excluded from the study with the presumption of viral etiology as the CBC and/or inflammatory markers were inconclusive. These patients also demonstrated a self-limiting clinical course and were, hence, given only symptomatic care. The remaining 207 (61.6%) patients had clinical features, inflammatory markers, and FNAC showing reactive changes favoring a diagnosis of bacterial lymphadenitis. Out of the 207, nine (4.3%) patients were clinically unstable and were thereby excluded. A total of 198 patients were randomized into Group A (99 patients) and Group B (99 patients), but six patients from Group B were lost to follow-up, so in the final analysis, only a total of 192 were considered.

**Figure 1:**
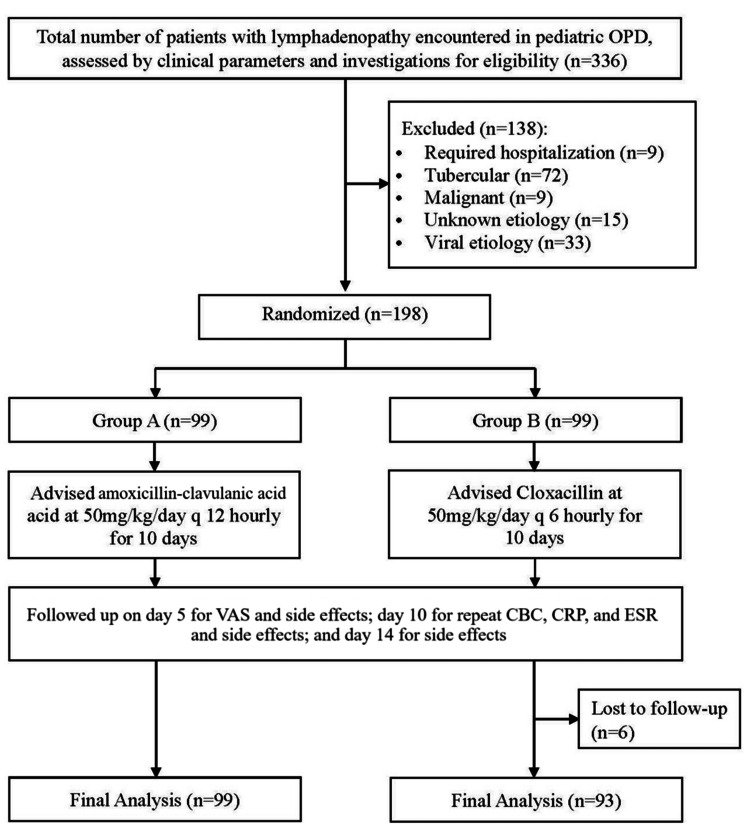
Flow of Recruitment of Participants in the Study OPD: outpatient department; VAS: visual assessment score; CBC: complete blood count; CRP: C-reactive protein; ESR: erythrocyte sedimentation rate

Figure 1 shows the process of recruitment of study subjects.

The spectrum of FNAC findings in the patients is presented in Table 1.

**Table 1:** Spectrum of FNAC Findings of Lymphadenopathy Cases FNAC: fine needle aspiration cytology

FNAC findings	Number of patients (n)	Percentage (%)
Reactive	240	71.43
Malignant	9	2.68
Tubercular	72	21.43
Inconclusive	15	4.46
Total	336	100.00

The mean (SD) age of the study participants was 7.48 (3.42) years. This included 75 patients in the age group 1-5 years and 117 patients in the age group 6-14 years. The mean (SD) age in Group A patients was 6.85 (2.74) years and in Group B was 7.27 (2.73) years with no significant difference (p = 0.55). Our study participants comprised 123 male patients and 69 female patients with a ratio of 1.78:1. The baseline age and gender distribution were comparable across both groups. The baseline characteristics of both groups as demonstrated in Table 2 were similar and comparable.

**Table 2:** Baseline Demographic Characteristics of Both Groups Before Treatment Data presented as n (%) or mean (SD). The chi-squared test is used to calculate the p-value for categorical data. SD: standard deviation; ꭓ^2^: chi-squared value

Characteristics	Group A (n = 33)	Group B (n = 31)	Statistical test value	p-value
Age (1-5 years)	15 (45.45)	10 (32.26)	ꭓ^2^ = 0.2007	0.65
Age (6-14 years)	18 (54.55)	21 (67.74)		
Female	11 (33.33)	12 (38.71)	ꭓ^2^ = 0.8457	0.59
Male	22 (66.67)	19 (61.29)		

The presenting symptoms and signs were also uniform across both groups. Out of the 192 patients with bacterial lymphadenitis included, fever was the predominant symptom noted in all (100%) patients followed by pain noted in 159 (82.81%) patients. Lymphadenopathy involving the cervical group was the most common site affecting 123 (64.06%) children. The mean (SD) baseline VAS was 4.97 (1.71) in Group A and 4.84 (1.63) in Group B with no significant difference (p = 0.76). Our study population demonstrated a mean (SD) baseline leukocytosis with Group A having a leukocyte count of 12.04 (2.15) x 10^9^/L and Group B 11.87 (2.28) x 10^9^/L (p = 0.78). We also noted an elevated ESR of 15.98 (3.31) mm/h and CRP of 17.49 (3.99) mg/L at the time of presentation. There was no significant difference in values between both groups as shown in Table 3.

**Table 3:** Comparison of Symptoms, Signs, and Investigations in Both Groups Before Treatment Data presented as n (%) or ^a^mean (SD). The chi-squared test is used to calculate the p-value for categorical data, and the t-test is used to calculate p-value for continuous data. SD: standard deviation; VAS: visual assessment score; CRP: C-reactive protein; ESR: erythrocyte sedimentation rate; ꭓ^2^: chi-squared value; t: t-test value

Signs, symptoms, and investigations	Group A (n = 99)	Group B (n = 93)	Statistical test value	p-value
Pain	75 (75.7)	84 (90.3)	ꭓ^2^ = 0.882	0.92
Fever	99 (100)	93 (100)		
Local rise in temperature	3 (3.3)	9 (10)	ꭓ^2^ = 3.775	0.43
Lymph node tenderness	60 (60.6)	69 (71.3)	ꭓ^2^ = 0.020	0.89
Size^a^ (mm)	17.3 (3.56)	17.01 (2.4)	t = 0.3257	0.74
VAS^a^	4.9 (1.71)	4.94 (1.63)	t = 0.310	0.75
Leukocytes^a^ (x10^9^/L)	12.04 (2.15)	11.87 (2.28)	t = 0.297	0.77
Neutrophils^a^ (%)	69.90 (4.83)	69.87 (3.77)	t = 0.920	0.37
ESR^a^ (mm/h)	16.70 (2.93)	15.23 (3.58)	t = 1.802	0.07
CRP^a^ (mg/L)	16.85 (4.33)	18.17 (3.54)	t = 1.330	0.19

Following treatment, our study revealed a combined recovery in 171 (89.07%) patients in the form of an improvement in fever. The mean defervescence time for Group A was 5.92 days (95% confidence interval (CI): 5.15 to 6.69 days) and for Group B was 5.74 days (95% CI: 4.96 to 6.52 days). Based on previous studies, 0.5 days was defined as the non-inferiority margin [9,10]. The upper bound of 95% CI in Group B (6.52) was only 0.5 days longer than the mean of Group A (5.92). This finding suggests the non-inferiority of cloxacillin in comparison with amoxicillin-clavulanic acid. We also calculated the median (IQR) defervescence time for Group A (five days, IQR: 4-7 days) and for Group B (five days, IQR: 4-7 days), which showed no statistically significant difference (p = 0.48). The 95% CI for the difference in fever rates (Group A - Group B) is -19.16% to +11.54%. Since the interval includes 0, there is no statistically significant difference in fever rates between the groups at the 95% confidence level indicating non-inferiority of cloxacillin.

As the primary outcome variable in our study was defervescence, we also carried out an intention-to-treat analysis by including the six patients who were lost to follow-up in Group B. The mean defervescence in Group A was 5.88 (95% CI: 5.11 to 6.65 days) and for Group B 5.79 (95% CI: 5.79 to 6.52 days). The calculated median (IQR) defervescence for Group A (five days, IQR: 4-7 days) and for Group B (five days, IQR: 4-7 days) also showed no statistically significant difference (p = 0.79).

In due course of treatment, the VAS significantly decreased in both the treatment groups with mean (SD) VAS of 1.09 (1.05) in Group A and 1.03 (0.74) in Group B after five days of treatment although the difference between the groups was comparable (p = 0.79). Post-treatment pain persisted in 27 (14.06%) and fever in 21 (10.93%) patients in both groups combined. These persistent symptoms were also distributed parallelly in both treatment groups (p = 0.99).

A demonstrable decline was noted following treatment in leukocyte count (Group A 9.83 (1.10) x 10^9^/L and Group B 9.95 (1.46) x 10^9^/L, p = 0.72), ESR (Group A 9.12 (2.18) mm/h and Group B 8.45 (2.38) mm/h, p = 0.26), and CRP (Group A 6.02 (1.49) mg/L and Group B 6.07 (1.53) mg/L, p = 0.90). Table 4 summarizes the comparison of symptoms, signs, and investigations observed in both groups after the completion of treatment.

**Table 4:** Symptoms, Signs, and Investigations in Both Groups After Treatment Data presented as n (%) or ^a^mean (SD). The chi-squared test is used to calculate the p-value for categorical data, and the t-test is used to calculate the p-value for continuous data. SD: standard deviation; VAS: visual assessment score; CRP: C-reactive protein; ESR: erythrocyte sedimentation rate; ꭓ^2^: chi-squared value; t: t-test value

Signs, symptoms, and investigations	Group A (n = 99)	Group B (n = 93)	Statistical test value	p-value
Pain	12 (12.1)	15 (16.1)	ꭓ^2^ = 0.054	0.99
Fever	9 (9.1)	12 (12.9)		
Local rise in temperature	0 (0)	3 (3.3)	ꭓ^2^ = 2.834	0.59
Lymph node tenderness	15 (15.1)	21 (22.5)	ꭓ^2^ = 0.395	0.52
Size^a^ (mm)	6.76 (1.88)	6.94 (1.54)	t = 0.417	0.68
VAS^a^	1.09 (1.05)	1.03 (0.74)	t = 0.262	0.80
Leukocytes^a^ (x10^9^/L)	9.83 (1.10)	9.95 (1.46)	t = 0.359	0.72
Neutrophils^a^ (%)	63.32 (4.45)	64.4 (4.11)	t = 0.985	0.32
ESR^a^ (mm/h)	9.12 (2.18)	8.45 (2.38)	t = 1.137	0.27
CRP^a^ (mg/L)	6.02 (1.49)	6.07 (1.53)	t = 0.128	0.90

**Figure 2:**
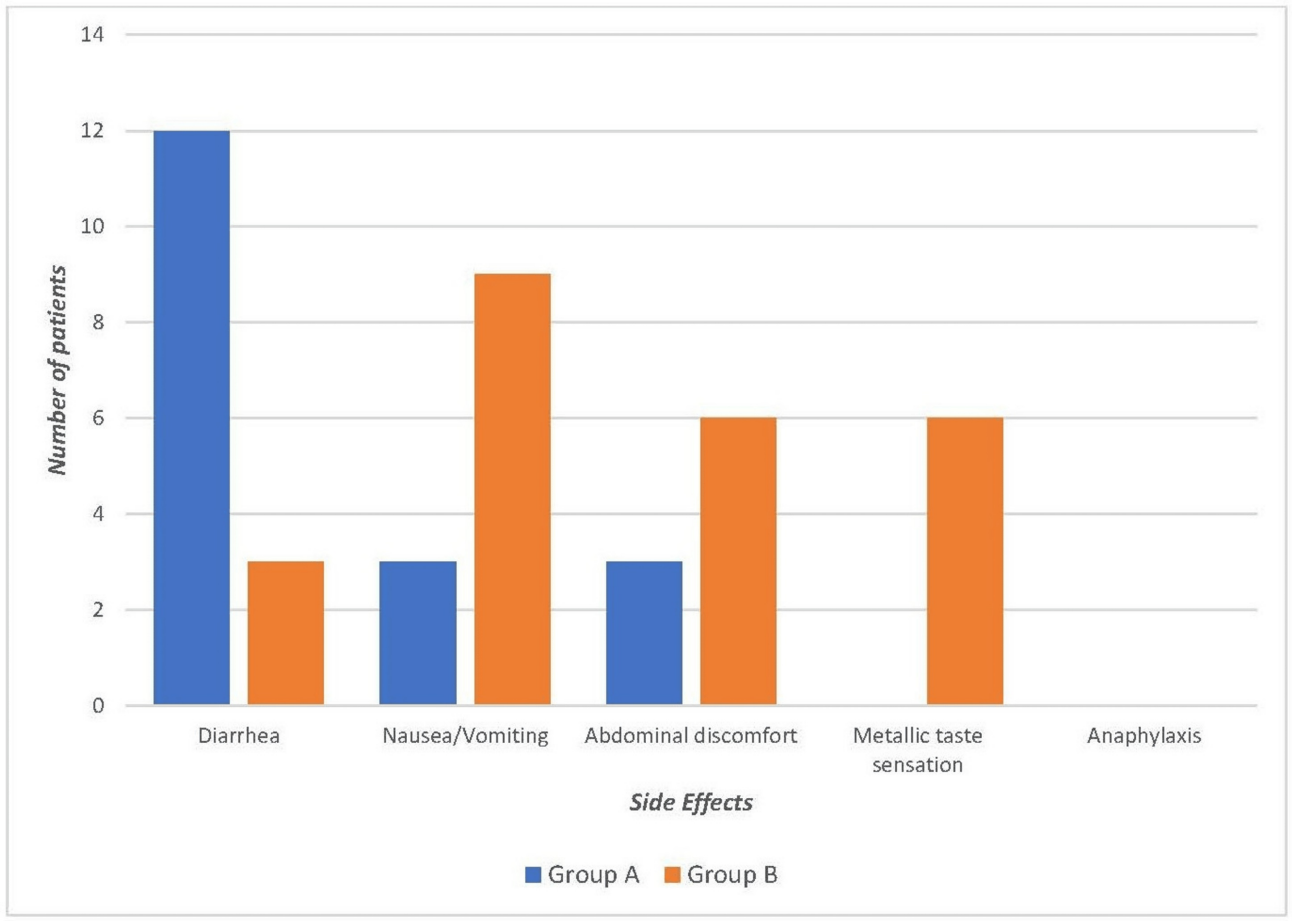
Comparison of Various Adverse Effects Between the Two Groups

The most common side effect encountered was diarrhea and nausea/vomiting in Group A and Group B, respectively. No adverse effects needing drug withdrawal were observed in the groups. There was no significant difference in the side effect profile of both drugs. Figure 2 summarizes the side effects observed in both groups.

## Discussion

Pediatric bacterial lymphadenitis often requires timely and appropriate antibiotic therapy to prevent complications. Amoxicillin-clavulanic acid, a broad-spectrum antibiotic, is widely used but carries a risk of promoting antibiotic resistance [11]. In contrast, cloxacillin, a narrower-spectrum antibiotic, targets staphylococcal infections more specifically and may offer a safer and equally effective alternative [12]. This study aims to determine whether cloxacillin is non-inferior to the efficacy of amoxicillin-clavulanic acid in treating bacterial lymphadenitis in children.

The mean (SD) age of the study participants was 7.48 (3.42) years, and the male-to-female ratio was 1.78:1. In a study conducted by Ataş et al., the median age and mean age of study participants were six years and 6.1 years, respectively [13]. The male-to-female ratio was 2.38:1, showing a male predominance like that of our study.

Fever was the most common presenting complaint noted in all of our participants, i.e., 192 (100%), followed by pain noted in 159 (82.81%) patients. The cervical group of lymph nodes was involved in 123 (64.06%) out of 192 patients. These findings from our study were akin to those of Rao et al., Reddy et al., and Mohan et al. where fever was the most common associated symptom and the cervical group was the most common site of lymph node enlargement [14-16].

On the basis of a study conducted by Howard-Jones et al., which showed that significantly elevated white cell counts, CRP, and ESR at baseline and on follow-up were associated with complicated lymphadenitis [17], we incorporated these inflammatory markers in our study to enable the diagnosis and judge the response to therapy in bacterial lymphadenitis. In our study, FNAC was used as a modality to rule out tubercular and malignant etiology for lymphadenopathy as it is sensitive, easily available, and minimally invasive. In a setting of acute or subacute onset lymphadenitis with clinical features and laboratory markers favoring a bacterial etiology, an FNAC showing reactive changes is highly contributory in establishing an early diagnosis even before microbiological evidence in the form of a culture can be obtained. High sensitivity rates of FNAC were demonstrated in many studies like that of Stansfield and Ardenne [18].

As our primary outcome variable was the day of defervescence, we calculated the recovery rate of our participants in terms of recovery from fever. An overall recovery rate of 89.07% was seen with a total of 171 out of 192 patients (Group A: 90 (90.91%) and Group B: 81 (87.1%)) demonstrating recovery from fever. Analogous to our study, Sarsu and Sahin reported a regression of symptoms and signs in 70.88% of cases [19]. In due course of treatment, the VAS significantly decreased in both the treatment groups with mean (SD) VAS of 1.09 (1.05) in Group A and 1.03 (0.74) in Group B after five days of treatment indicating pain relief, although the difference between the groups was comparable (p = 0.79).

Gastrointestinal tract-related complaints were the most common side effects encountered with both antibiotics. These findings were consistent with those of Matsui et al. and Kar et al. wherein tolerable gastrointestinal symptoms were the predominant side effects of both drugs [20-23]. We noticed that the compliance in the cloxacillin group was lower due to four times daily dosage and its associated metallic taste; this finding in our study is consistent with that noted in the literature on the side effect profile of cloxacillin [20,21].

As this is a single-center study with a small sample size, the generalizability of its findings is limited. Also, potential confounding factors such as concurrent infections or underlying health conditions that might influence treatment outcomes were not considered. Hence, it is recommended to conduct larger, multicenter randomized controlled trials to confirm the findings and enhance the generalizability of the results of this study. A more comprehensive assessment of nutritional status and dietary evaluations would provide a clearer understanding of the impact of antibiotic treatment on nutritional recovery in pediatric patients.

## Conclusions

To conclude, the above findings suggest that both antibiotics are viable options for managing pediatric bacterial lymphadenitis, with the choice of treatment potentially guided by the availability of the drugs and the clinician’s choice.

Overall, the study contributes valuable insights into the management of pediatric bacterial lymphadenitis, reaffirming the efficacy of both amoxicillin-clavulanic acid and cloxacillin as effective therapeutic options in clinical practice. Cloxacillin being a narrower-spectrum antibiotic as compared to amoxicillin-clavulanic acid, can be an equivalent choice, potentially conferring the benefit of curbing the surging antibiotic resistance.
